# Supplementary material for: De Novo Atomic Protein Structure Modeling for Cryo-EM Density Maps Using 3D Transformer and Hidden Markov Model
Source: bioRxiv. 2024 Jan 2:2024.01.02.573943. Preprint. [Version 1] doi: 10.1101/2024.01.02.573943 (PMC10802328; doi:10.1101/2024.01.02.573943)
Supplement: 1 [file NIHPP2024.01.02.573943V1-supplement-1.pdf]

bioRxiv preprint doi: https://doi.org/10.1101/2024.01.02.573943; this version posted January 2, 2024. The copyright holder for this preprint (which was not certified by peer review) is the author/funder, who has granted bioRxiv a license to display the preprint in perpetuity. It is made available under aCC-BY 4.0 International license.

Appendix A Supplementary Data

bioRxiv preprint doi: https://doi.org/10.1101/2024.01.02.573943; this version posted January 2, 2024. The copyright holder for this preprint (which was not certified by peer review) is the author/funder, who has granted bioRxiv a license to display the preprint in perpetuity. It is made available under aCC-BY 4.0 International license.

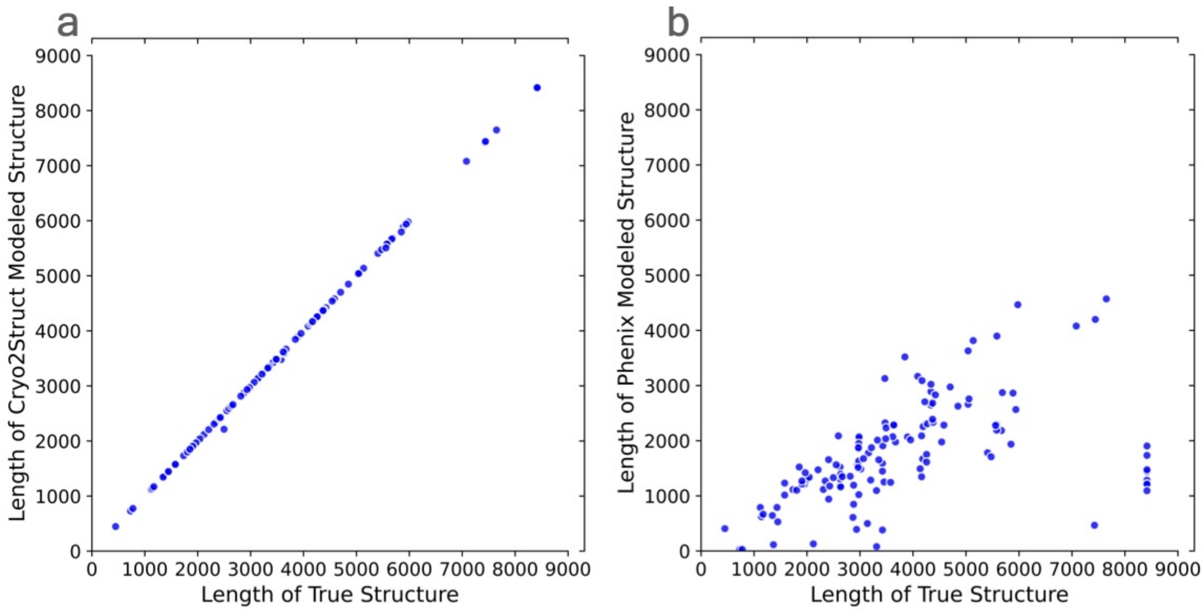

Fig. A1 Length of structural models constructed by Cryo2Struct or Phenix versus (VS) length of the true structures in the standard test dataset. (**a**) Cryo2Struct models VS true structures. (**b**) Phenix models VS true structures.

bioRxiv preprint doi: https://doi.org/10.1101/2024.01.02.573943; this version posted January 2, 2024. The copyright holder for this preprint (which was not certified by peer review) is the author/funder, who has granted bioRxiv a license to display the preprint in perpetuity. It is made available under aCC-BY 4.0 International license.

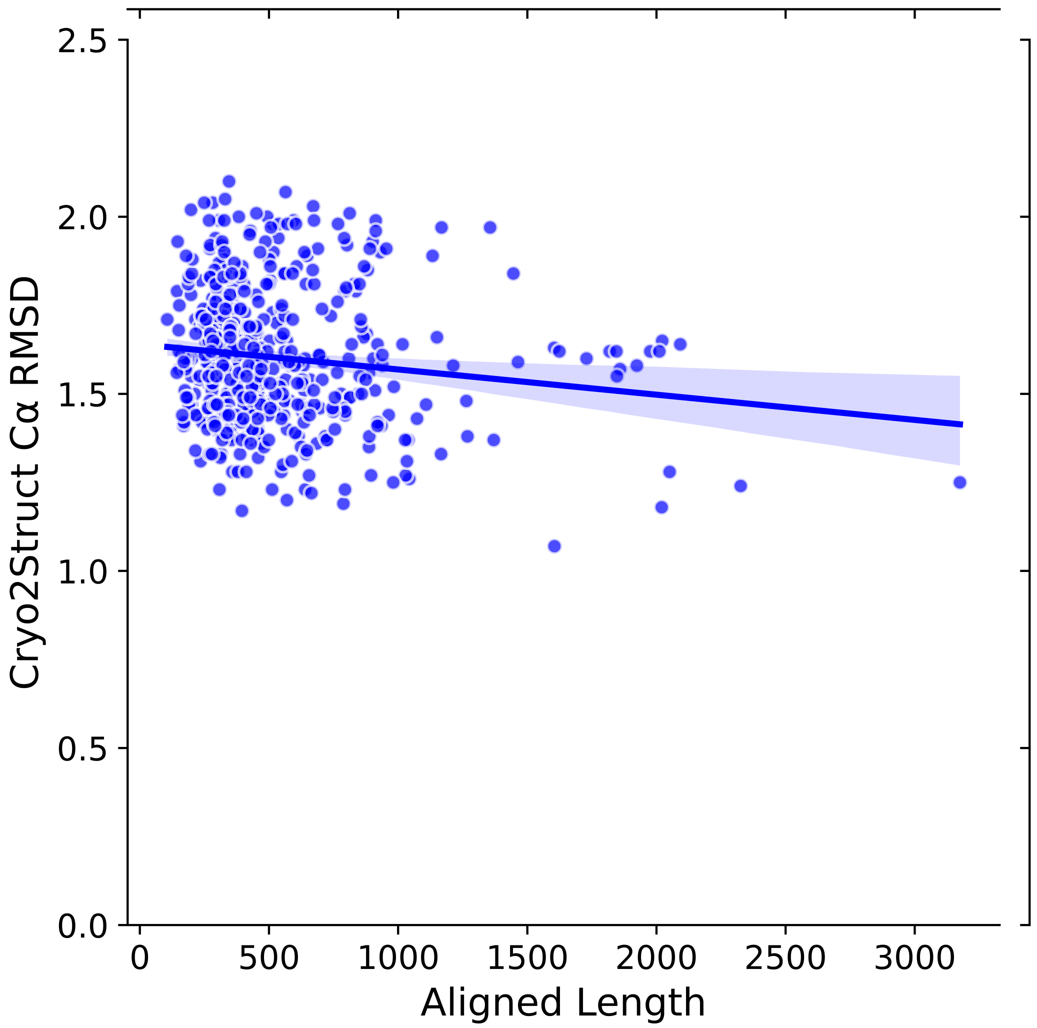

Fig. A2 RMSD versus the length of the aligned regions of the atomic models built for 500 test cryo-EM maps. The models were aligned with the true structuers by US-align. The solid line depicts linear regression line, and the colored area represents a 95% confidence interval. The regression equation: $y = -0.0001x + 1.6401$; the correlation: -0.134. The average RMSD of the models is 1.60 Å. The average aligned length is 532.51 where as the average length of true structure is 1837.43. Cryo2Struct models have about 29% aligned length.

bioRxiv preprint doi: https://doi.org/10.1101/2024.01.02.573943; this version posted January 2, 2024. The copyright holder for this preprint (which was not certified by peer review) is the author/funder, who has granted bioRxiv a license to display the preprint in perpetuity. It is made available under aCC-BY 4.0 International license.

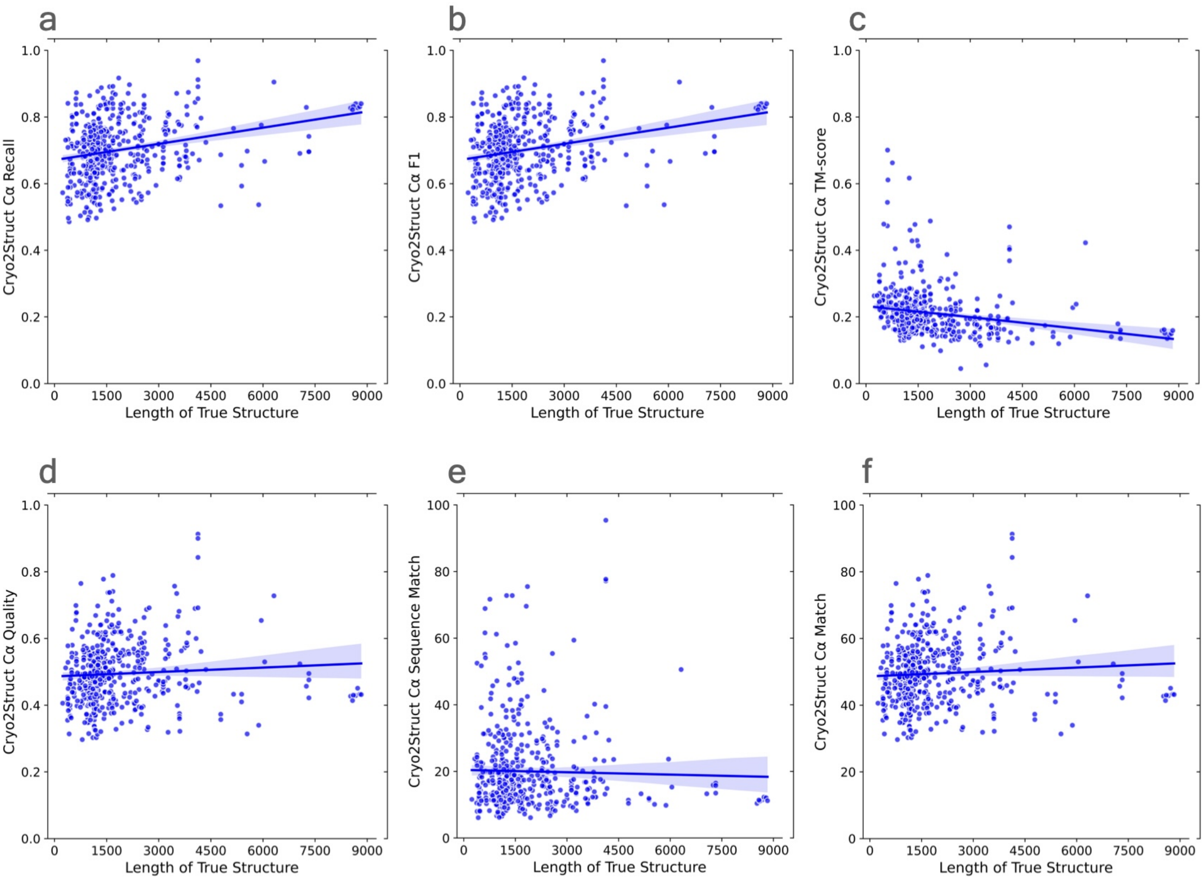

Fig. A3 The quality scores of atomic models built for the 500 cryo-EM maps in the new test dataset versus (VS) the length of the true structures. The solid lines depicts linear regression lines, and the colored area represents a 95% confidence interval. (**a**) The Cα recall VS length of true structure; the regression equation: $0.0000x + 0.6712$; Pearson's correlation: 0.259. (**b**) The F1 score VS length of true structure; the regression equation: $0.0000x+0.6714$; the correlation: 0.258. (**c**) The normalized TM-score VS length of true structure; the regression equation: $-0.0000x + 0.2328$; the correlation: -0.214. (**d**) The Cα quality score VS length of true structure; the regression equation: $0.0000x + 0.4863$; the correlation: 0.066. (**e**) The Cα sequence match score VS length of true structure; the regression equation: $-0.0002x + 20.4579$; the correlation: -0.025. (**f**) The Cα match score VS length of true structure; the regression equation: $0.0004x + 48.6615$; the correlation: 0.065.

bioRxiv preprint doi: https://doi.org/10.1101/2024.01.02.573943; this version posted January 2, 2024. The copyright holder for this preprint (which was not certified by peer review) is the author/funder, who has granted bioRxiv a license to display the preprint in perpetuity. It is made available under aCC-BY 4.0 International license.

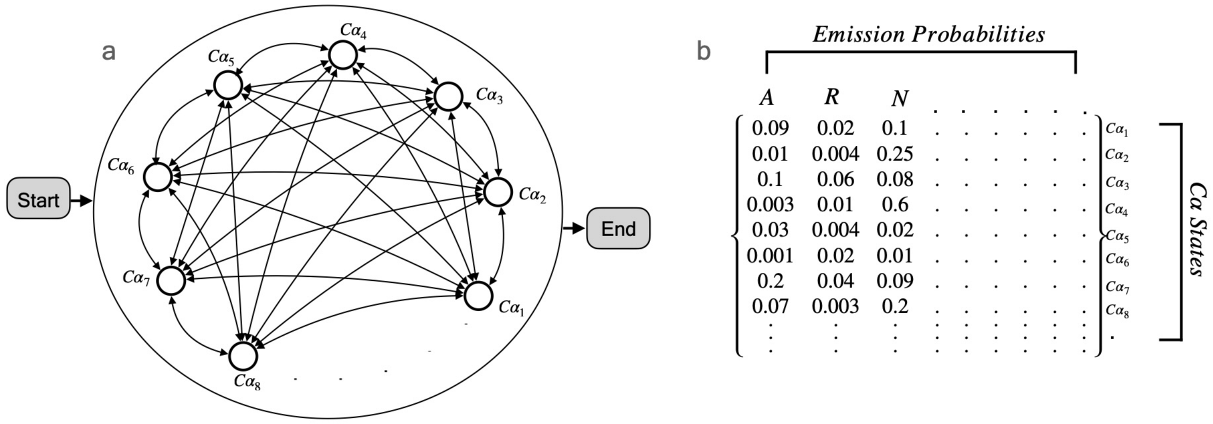

Fig. A4 A Hidden Markov Model (HMM) used for aligning protein sequences with predicted Cα atoms (voxels) to generate protein backbone traces. **(a)** The states of the fully connected HMM. A hidden path can start from or end at any Cα state. It is worth noting that there is no gap state in the HMM and therefore every amino acid in a protein sequence can be aligned to one Cα atom. **(b)** The emission probabilities of the hidden Cα states are the normalized geometric mean of the predicted amino acid type probability and the background (prior) probability for 20 amino acids in the nature, referred to by their abbreviation.
